# Exploration of the Pathogenesis of Chronic Obstructive Pulmonary Disease Caused by Smoking—Based on Bioinformatics Analysis and In Vitro Experimental Evidence

**DOI:** 10.3390/toxics11120995

**Published:** 2023-12-07

**Authors:** Yingchi Zhang, Yuxin Sheng, Yanrong Gao, Yujia Lin, Bin Cheng, Hongmei Li, Ling Zhang, Haiming Xu

**Affiliations:** 1 School of Public Health, Ningxia Medical University, Yinchuan 750004, Chinashengyuxin1001@163.com (Y.S.); gaoyanrong726@163.com (Y.G.); lyj1056874570@163.com (Y.L.); lihongmei@nxmu.edu.cn (H.L.); 2 The Key Laboratory of Environmental Factors and Chronic Disease Control, Yinchuan 750004, China; 3 Xi’an Center for Disease Control and Prevention, Xi’an 710000, China; 4 The Key Laboratory of Fertility Preservation and Maintenance of the Ministry of Education, Ningxia Medical University, Yinchuan 750004, China; 5 School of Basic Medicine, Ningxia Medical University, Yinchuan 750004, China

**Keywords:** smoking, COPD, Go analysis, KEGG analysis, protein interaction network, miRNA, validation

## Abstract

This study was aimed at investigating the pathogenesis of chronic obstructive pulmonary disease (COPD) caused by smoking—based on bioinformatics analysis and in vitro experimental evidence. The GEO, GEO2R, TargetScan, miRDB, miRWalk, DAVID, and STRING databases were used for bioinformatics analysis. The mRNA expression and the protein levels were determined by real-time PCR and ELISA. After taking the intersection of the diversified results of the databases, four differentially expressed miRNAs (hsa-miR-146a, hsa-miR-708, hsa-miR-150, and hsa-miR-454) were screened out. Subsequently, a total of 57 target genes of the selected miRNAs were obtained. The results of DAVID analysis showed that the selected miRNAs participated in COPD pathogenesis through long-term potentiation, the TGF-β signaling pathway, the PI3K-Akt signaling pathway, etc. The results of STRING prediction showed that TP53, EP300, and MAPK1 were the key nodes of the PPI network. The results of the confirmatory experiment showed that, compared with the control group, the mRNA expression of *ZEB1*, *MAPK1, EP300,* and *SP1* were up-regulated, while the expression of MYB was down-regulated and the protein levels of ZEB1, MAPK1, and EP300 were increased. Taken together, miRNAs (hsa-miR-146a, hsa-miR-708, hsa-miR-150, and hsa-miR-454) and their regulated target genes and downstream protein molecules (ZEB1, EP300, and MAPK1) may be closely related to the pathological process of COPD.

## 1. Introduction

Smoking is a major public health issue worldwide, with significant impacts on human health and socio-economic development. It is estimated that the global number of smoking-related deaths exceeds eight million annually, including both smokers themselves and passive smokers. Numerous studies have shown that cigarette smoke contains a large amount of inhalable particulate matter (IPM), which is the main source of IPM in indoor environments [1,2]. For example, PM2.5 levels could reach an average of 84 μg/m^3^ in the primary home smoking area [3]. Smoking is closely related to many serious health problems. Smoking causes various diseases, including lung cancer, cardiovascular disease, chronic obstructive pulmonary disease (COPD), and so on. The latest results from a prospective study on chronic diseases in China (CKB project) explored the disease burden caused by smoking in the Chinese population. The results showed that smoking is associated with the risk of various diseases and deaths among Chinese adults, and the burden of diseases caused by smoking will further increase in the future among males [4].

COPD is a common chronic respiratory disease characterized by persistent airflow limitation, which is related to increased chronic inflammatory response of airways and lungs to toxic particles or gases, with high disability and mortality [5]. COPD is the third leading cause of death globally, causing 3.23 million deaths in 2019. Smoking accounts for 70% and 30–40% of cases of COPD in high-income countries and low- and middle-income countries, respectively (data sourced from WHO official website).

There are many pathologic mechanisms leading to COPD, and the role of miRNA in this process has received much attention. MicroRNAs (MiRNAs) are one kind of non-coding single-stranded RNAs with a length of approximately 19–24 nt, which partially or completely complement the 3′-untranslated region (3′-UTR) of the target genes by complementary base pairing and then cut the transcripts of the target genes or inhibit the translation of the transcripts, thus directly regulating the functional expression of the target genes after transcription. MiRNAs are involved in many biological processes, including body development and cell differentiation, proliferation, and apoptosis, as well as inflammatory response and the pathological processes of tumors and other diseases [6]. For example, Ouyang et al. found that miR-185-5p and miR-182-5p were involved in the development of COPD and might serve as potential biomarkers for the diagnosis of COPD [7]. Ding et al. also found that miR-150-5p could be used for the diagnosis and disease assessment of COPD [8]. The expression levels of miR-203a-3p and miR-375 were significantly higher in both COPD patients and healthy people with smoking [9]. Fang et al.’s research indicated that miR-101-3p was closely related to the occurrence of COPD, and that blocking it might provide a new method for the treatment of COPD [10]. In recent years, researchers have begun to explore the association between genes, signaling pathways, biological effects regulated by miRNAs, and the pathological process of COPD. For instance, Zhu et al.’s research suggested that miR-126-5p-MYC and miR-130b-5p-FOXO1 might be used as biomarkers for the diagnosis and treatment of COPD patients [11]. Shen et al. reported that miR-221-3p and miR-92a-3p mediated inflammatory disorders that were associated with COPD [12]. In summary, miRNAs indeed mediate the pathological process of COPD with diverse roles. However, objectively speaking, there is still a long way to go before fully revealing the roles of miRNAs and their regulatory molecular networks in the pathogenesis of COPD.

In this study, a combination of database mining and experimental verification methods was employed to reveal the role of miRNAs and their regulated gene protein networks in smoking-induced COPD at the overall level. The results of this study can provide scientific reference for the pathogenesis research, clinical diagnosis, and treatment of COPD.

## 2. Materials and Methods

### 2.1. Data Retrieval

Taking GEO (https://www.ncbi.nlm.nih.gov/gds/, accessed on 6 December 2022) as the target database, “miRNA” and “COPD” as the keywords, “*Homo sapiens*” as the species source, and “expression profiling by array” or “non-coding RNA profiling by array” as the type, the miRNA chip data related to COPD were retrieved. Based on the reference of relevant studies, the following data screening criteria were determined: Only the original experimental design corresponding to chip data met the specifications of the case-control study, and the number of tissue samples of case and control greater than or equal to 2 were included in the study. If the amount of filtered data was too large, the data were further filtered according to the sample size and data integrity. According to the above standard, 6 miRNA chip data were screened out, and 3 miRNA chip data with relatively large sample size and complete data containing COPD and control groups were selected (GSE61741, GSE38974, and GSE136390). To be specific, the GSE61741 data set included 9 COPD cases (GSM1512504–GSM1512509, GSM1512530–GSM1512532) and 2 control cases (GSM1512502 and GSM1512503), the GSE38974 data set included 8 COPD cases (GSM953120–GSM953127) and 19 control cases (GSM953128–GSM953146), and the GSE136390 data set included 7 COPD cases (GSM4047866–GSM4047872) and 14 control cases (GSM4047873–GSM4047886). GSE61741 and GSE136390 were derived from the peripheral blood samples of COPD and normal controls, and GSE38974 was derived from lung tissue samples of COPD patients who smoked and the normal control lung tissue samples of healthy smokers.

### 2.2. Screening of COPD-Related Differentially Expressed miRNA and Prediction of Target Genes

The GEO2R online analysis tool (https://www.ncbi.nlm.nih.gov/geo/geo2r/, accessed on 13 December 2022) was used in the screening of microRNAs associated with COPD chip differentially expressed microRNAs screening criteria for │log2(FC)│ > 1 and *p*.Val < 0.05 or ad*p*.Val < 0.05. The online tool (https://www.cloudtutu.com, accessed on 13 December 2022) was used to draw volcano plots and cluster heat maps. The intersection of differentially expressed miRNAs was obtained from 3 data samples (GSE61741, GSE38974, and GSE136390) using the online tool Venny 2.1 (https://bioinfogp.cnb.csic.es/tools/venny/index.html, accessed on 15 December 2022). Based on this, TargetScan (http://www.targetscan.org/, accessed on 18 December 2022), miRDB (http://www.mirdb.org/, accessed on 18 December 2022), and MiRWalk (http://mirwalk.umm.uni-heidelberg.de/search-miRNAs/, accessed on 18 December 2022) were used to predict mRNAs regulated by differentially expressed miRNAs [13,14,15]. 

### 2.3. Analysis of Functional Enrichment of Target Genes Regulated by COPD-Related miRNAs

The Database for Annotation, Visualization, and Integrated Discovery (DAVID) (https://david.ncifcrf.gov/, accessed on 19 December 2022) was used for analyzing the function of enrichment of miRNA target genes [11]. The database included gene ontology (GO) analysis and pathway enrichment analysis of the Kyoto Encyclopedia of Genes and Genomes (KEGG) database. GO analysis included biological process (BP), cellular component (CC), and molecular function (MF).

### 2.4. Construction of Interaction Network of Target Gene Proteins Regulated by COPD-Related miRNAs

The online tool STRING (https://string-db.org/, accessed on 20 December 2022) was used for protein interaction analysis of predicted target genes, and a protein–protein interaction (PPI) network was constructed to obtain the key nodes [16].

### 2.5. Experimental Validation of Target Genes Regulated by COPD-Related miRNAs

#### 2.5.1. Cell Culture 

Fetal bovine serum (10%) and penicstreptomycin (1%) were added into DMEM basal medium as a complete culture medium for human lung adenocarcinoma A549 cells (TCHu150, Cell Bank of Chinese Academy of Sciences, Shanghai, China). The cells were cultured in a 5% carbon dioxide incubator at 37 °C. Trypsin containing 0.01% EDTA was used for digestion and passage.

#### 2.5.2. Construction of COPD Cell Model 

A549 cells were exposed to 5% cigarette smoke extracts (CSE) for 48 h. CSE raw liquid was prepared by suction filter method [17,18]. Briefly, the smoke of 10 standard cigarettes (84 mm × 8 mm, nicotine content: 1.5 mg/tablet, tar content: 15 mg/tablet) was continuously collected with a smoke collection device. Then, it was dissolved in 50 mL serum-free DMEM basic medium. A 0.22 μm membrane filter was used for the sterilizing filtration. The filtered solution was defined as 100% CSE stock solution. Referring to the relevant research and our pre-experimental results, the concentration of working solution in the subsequent experiment was 5%. The theoretical concentration of nicotine in 5% CSE working solution was 0.015 mg/mL.

#### 2.5.3. Determination of mRNA Expression Levels 

The total RNA in A549 cells was extracted by a total RNA extraction kit (Tiangen Biotech, Beijing, China), and the concentration of total RNA was measured with NanoDrop2000 (Thermo Scientific, Waltham, MA, USA). RNA was reverse transcribed into cDNA by a reverse transcription kit, and the relative expression of gene mRNA was measured by RT-PCR assays (Tiangen Biotech, Beijing, China) in a Light-Cycler 480 real-time PCR system (Roche, Basel, Switzerland). The above operation was carried out in strict accordance with the operating instructions of the commercial kits and *Molecular Cloning: A Laboratory Manual.* The primer sequences are shown in Table 1. *β-actin* was selected as the internal reference gene. The gene expression levels were analyzed by the classical 2^−ΔΔCT^ method.

#### 2.5.4. Determination of the Protein Contents 

ZEB1, MAPK1, and EP300 levels were determined by ELISA with commercial kits according to the product instructions (Dogesce, Beijing, China). The product codes for these three test kits are DG96287Q, DG96290Q, and DG96293Q, respectively. Before determining the protein concentrations in A549 cells after exposure to 5% CSE working solution, a reasonable dilution ratio was determined according to the preliminary experimental results to ensure that the determination results were within the linear determination range of the kits. According to the operation instructions of the corresponding kits, the OD values of standards and samples were measured at 450 nm with a microplate reader (SpectraMax i3x, Vienna, Austria).

### 2.6. Statistical Analysis

The experimental data were analyzed by SPSS 25.0 software, and the results were plotted using Graphpad Prism 7.0. The experimental data were expressed as mean and standard deviation (SD) and analyzed by independent sample T test and Dunnett’s T3 test; *p* < 0.05 was considered to be statistically significant and *p* < 0.01 was considered to be extremely statistically significant.

## 3. Results

### 3.1. Differentially Expressed miRNAs in COPD

**Figure 1:**
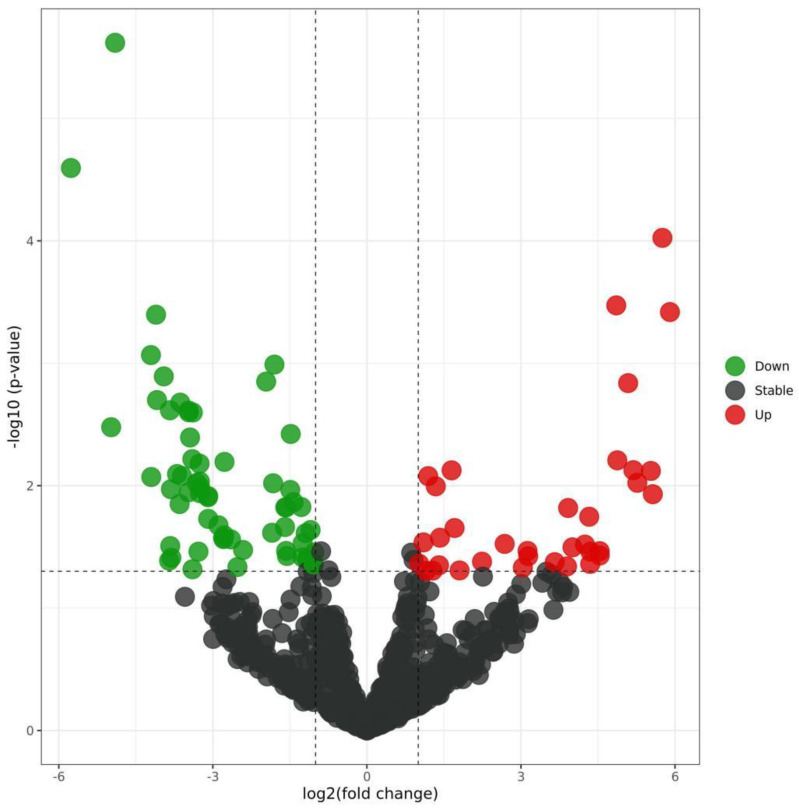
Volcano plot of GSE61741.

**Figure 2:**
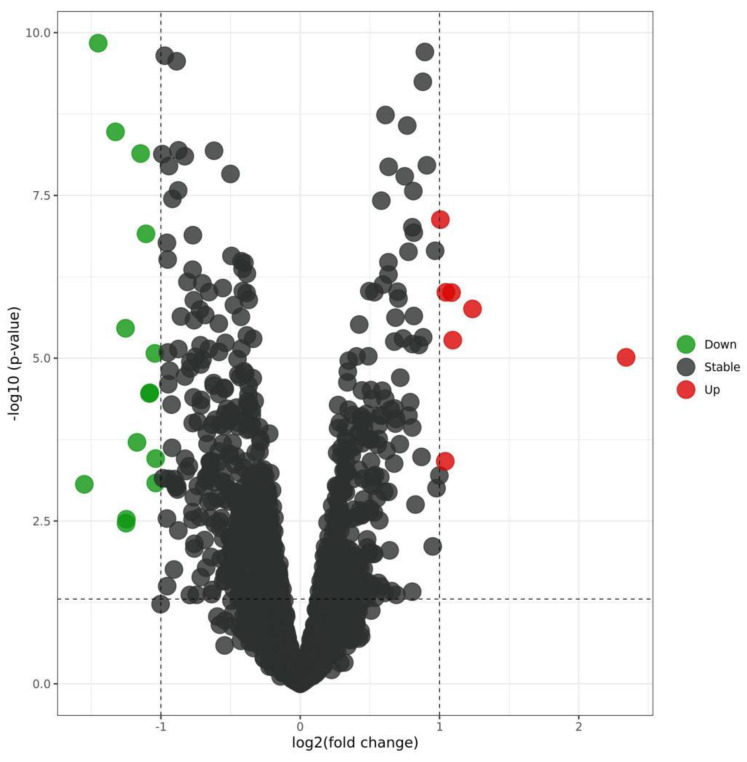
Volcano plot of GSE38974.

**Figure 3:**
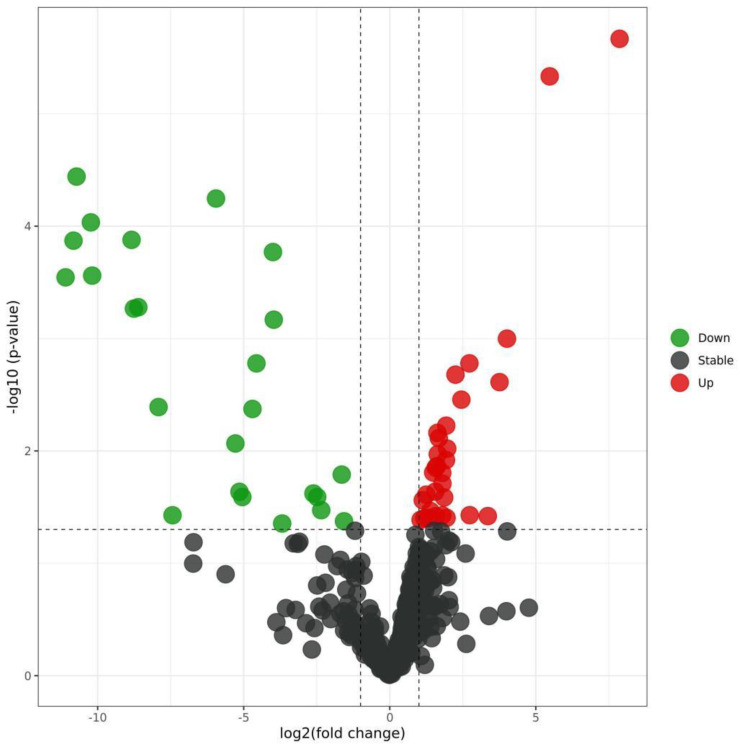
Volcano plot of GSE136390.

**Figure 4:**
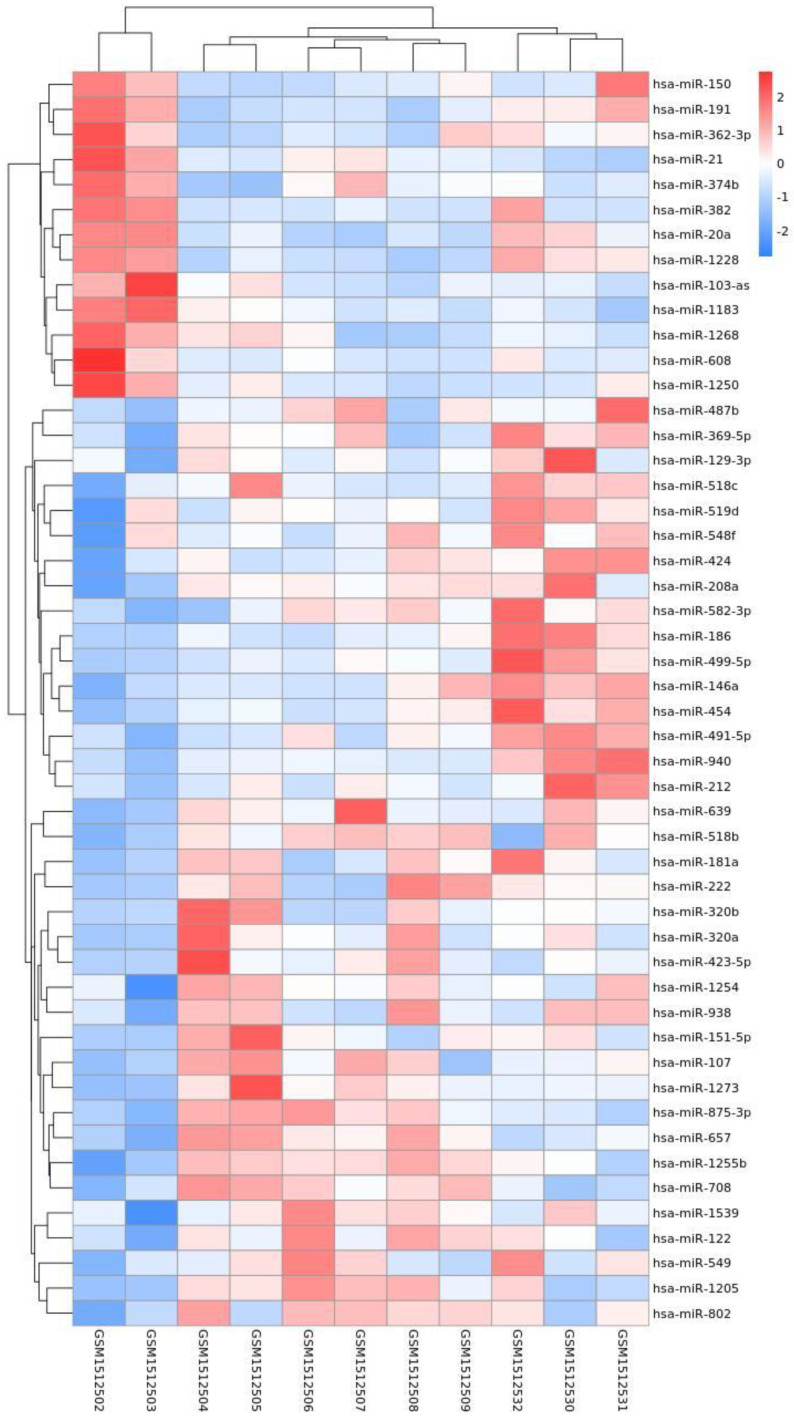
Heat map of GSE61741.

**Figure 5:**
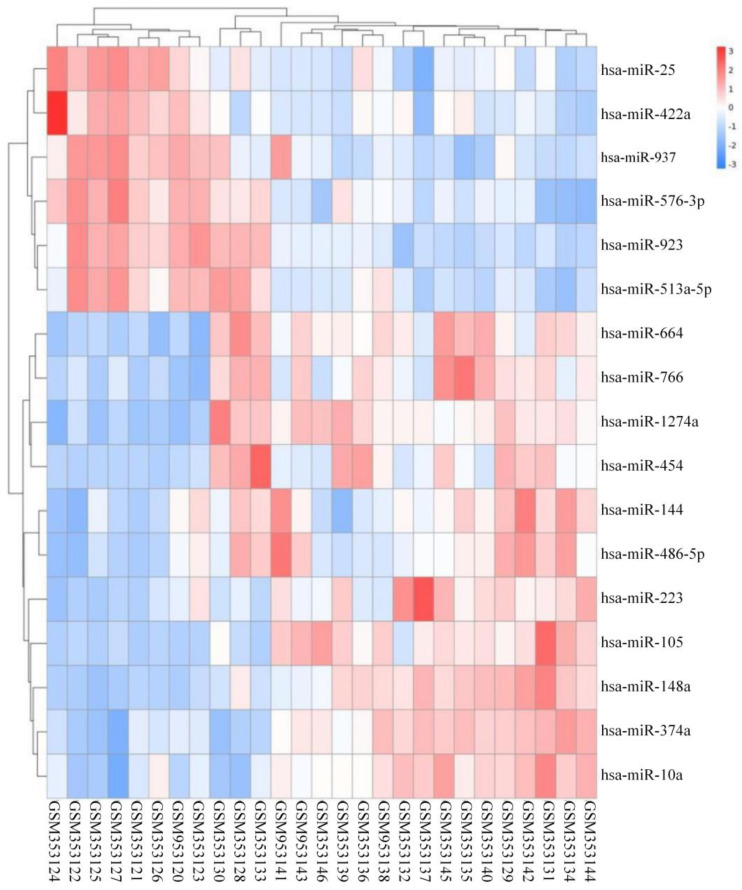
Heat map of GSE38974.

**Figure 6:**
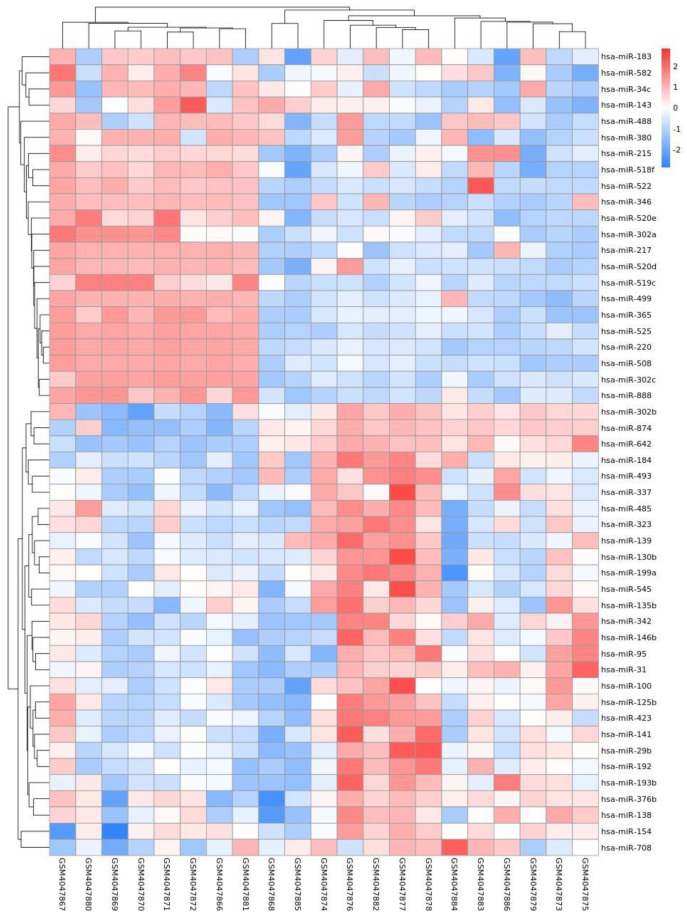
Heat map of GSE136390.

By analyzing the differential expression profiles of the samples from the subjects in the COPD and control groups, miRNAs with differential expression were screened. To be specific, 99 (64↓ vs. 35↑), 17 (11↓ vs. 6↑), and 53 (22↓ vs. 31↑) miRNAs with differential expression were screened from GSE61741, GSE38974, and GSE136390, respectively (Tables S1–S3). Volcano maps (Figure 1, Figure 2 and Figure 3) and heat maps (Figure 4, Figure 5 and Figure 6) showed that the screened miRNAs with differential expression had relatively good discrimination between the COPD group and the normal group.

### 3.2. Screening of COPD-Related Differentially Expressed miRNA and Prediction of Target Genes

**Figure 7:**
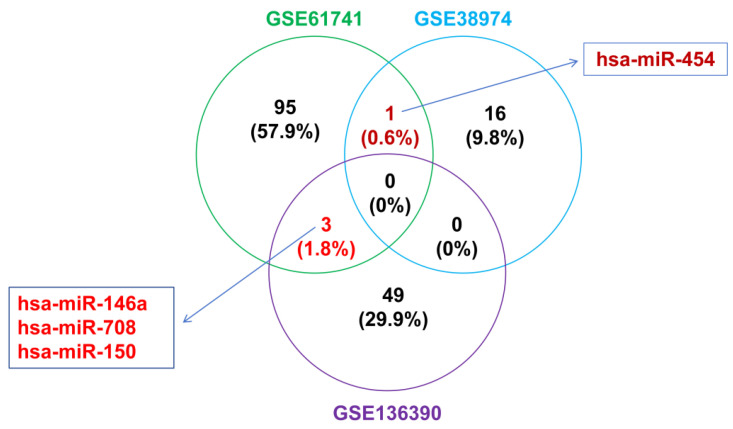
Venn diagram of the intersection of three miRNA microarray expression data sets (GSE61741, GSE38974, and GSE136390).

The intersection of differentially expressed miRNAs was taken as the miRNAs for the subsequent prediction of gene regulation, and four miRNAs were obtained, namely, hsa-miR-146a, hsa-miR-708, hsa-miR-150, and hsa-miR-454 (Figure 7). TargetScan, miRDB, and miRWalk were used to predict the target genes of the above miRNAs, respectively. A total of 57 differentially expressed miRNA target genes were obtained.

### 3.3. Functional Analysis Results of Target Genes with Differentially Expressed miRNAs Related to COPD

**Figure 8:**
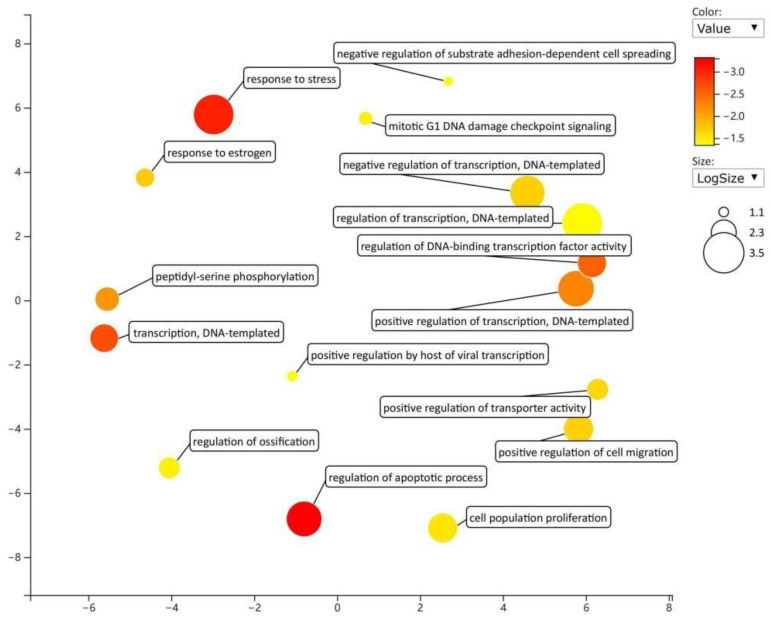
Enrichment items and diagrams of biological processes (BP).

**Figure 9:**
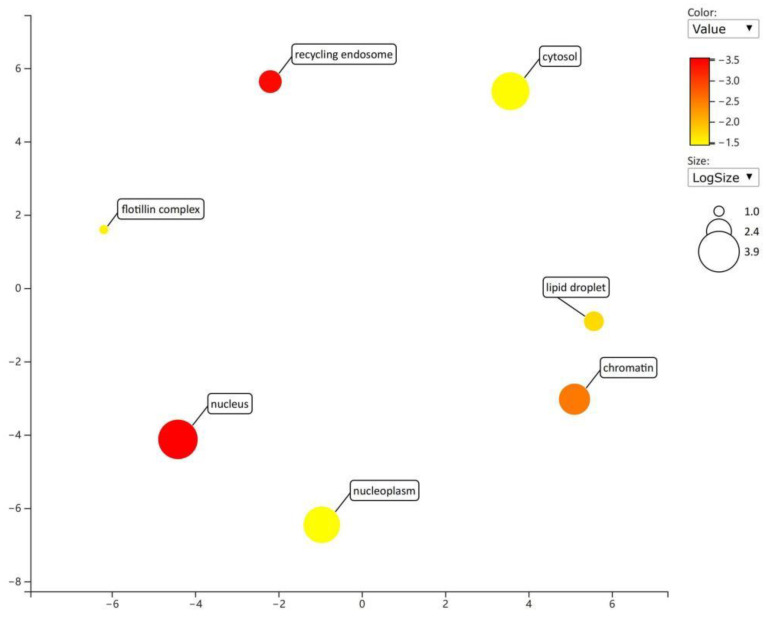
Enrichment items and diagrams of cell components (CC).

**Figure 10:**
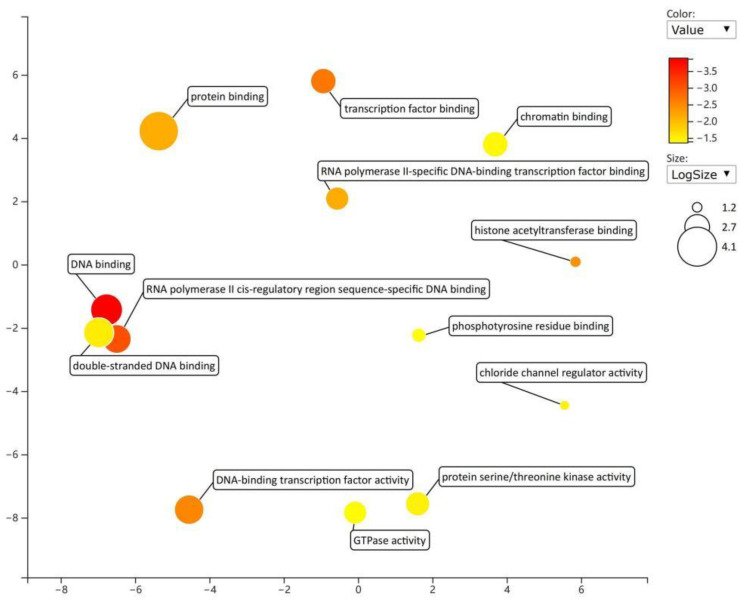
Enrichment items and diagrams of molecular function (MF).

**Figure 11:**
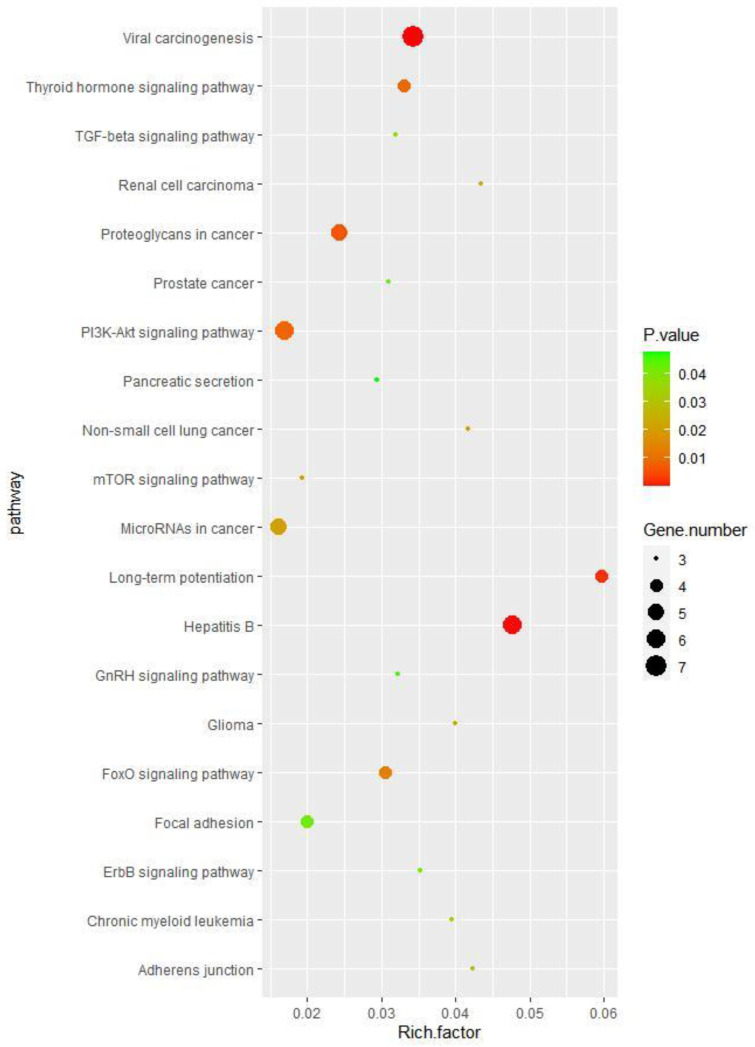
KEGG enrichment items of the target genes of the differentially expressed miRNAs associated with COPD.

Gene function enrichment analysis was performed on the target genes regulated by miRNAs in the regulatory network from the DAVID database. The enrichment items of biological processes mainly included the negative regulation of substrate adhesion-dependent cell spreading and the regulation of sequence-specific DNA binding transcription factor activity (Figure 8); the enrichment items of cell components mainly included lipid particles and cytoplasm (Figure 9); and the enrichment items of molecular function mainly included transcription factor activity, sequence specific DNA binding, and GTPase activity (Figure 10). KEGG pathway analysis showed that the first five enrichment items were long-term potentiation, hepatitis B, renal cell carcinoma, adherens junction, and non-small cell lung cancer (Figure 11).

### 3.4. PPI Network Diagram of COPD-Related miRNA Target Genes with Differential Expression

**Figure 12:**
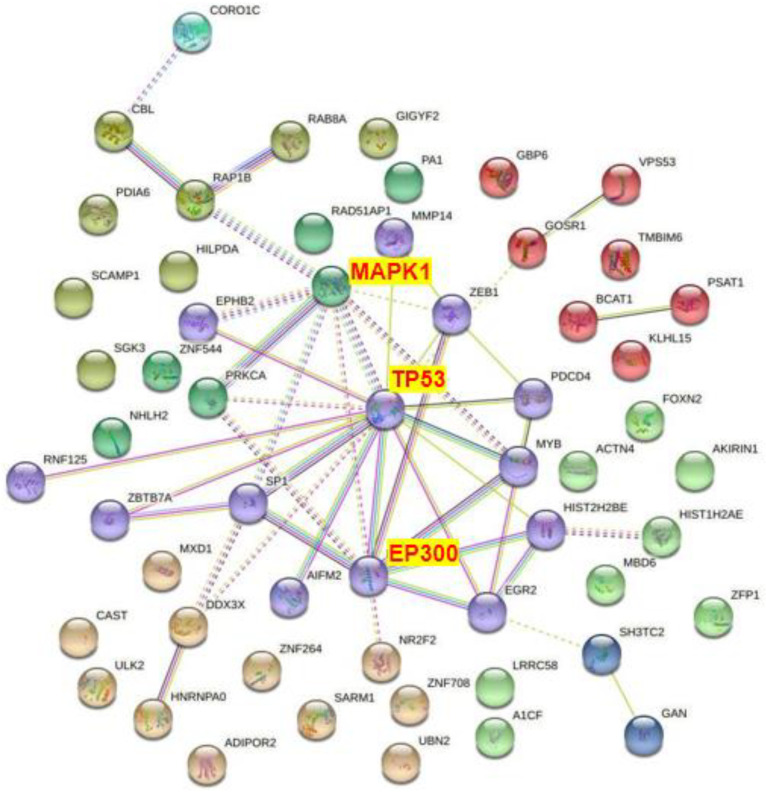
PPI network diagram of the target genes of the differentially expressed miRNAs associated with COPD.

After selecting the intersection of all mRNAs predicted by Targetscan, miRDB, and miRWalk, the protein interaction was predicted through the STRING database and the PPI network map was constructed. According to the PPI network diagram, TP53, EP300, and MAPK1 were associated with more than five groups of proteins. In addition, PRKCA SP1, EGR2, ZEB1, and MYB were also associated with other proteins (Figure 12). The quantitative data of PPI analysis are shown in Table S4. These results suggested that these proteins were at the key nodes of the PPI network and might play important roles in the pathological process of COPD.

### 3.5. Real-Time PCR Results

**Figure 13:**
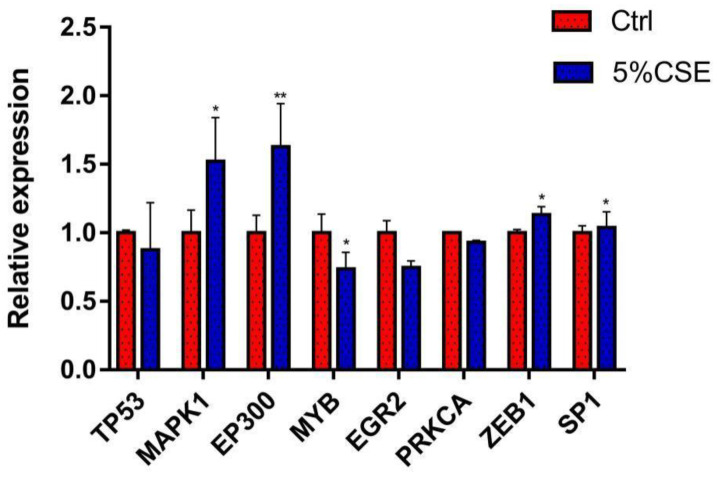
Gene expression levels after A549 cells were exposed to 5% cigarette smoke extracts (CSE) for 48 h. *n* = 3. * *p* < 0.05, ** *p* < 0.01.

Compared with the control group, exposure to 5% CSE working solution could lead to significant changes in the mRNA expression levels of the selected key molecules. Specifically, *ZEB1*, *MAPK1*, and *SP1* expression levels were significantly increased (*p* < 0.05); *EP300* level was extremely significantly increased (*p* < 0.01); while *MYB* level was significantly decreased (*p* < 0.05) (Figure 13).

### 3.6. ELISA Results

**Figure 14:**
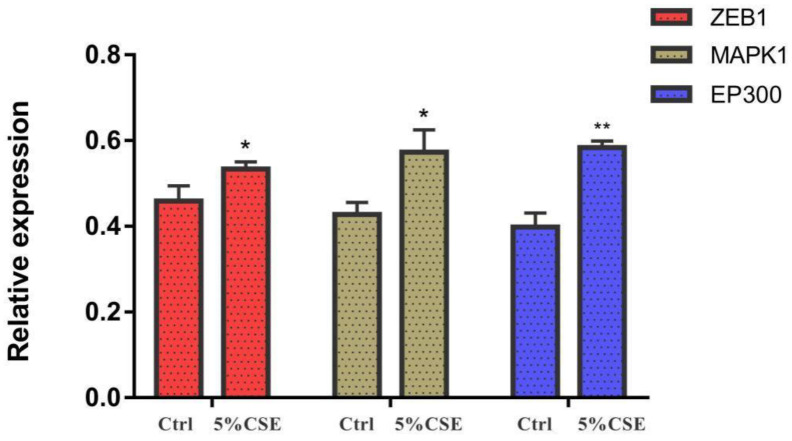
Protein expression levels after A549 cells were exposed to 5% cigarette smoke extracts (CSE) for 48 h. *n* = 3. * *p* < 0.05, ** *p* < 0.01.

CSE exposure could up-regulate the protein levels of ZEB1, MAPK1, and EP300 to a certain extent. To be specific, the protein levels of these three molecules in A549 cells after exposure to 5% CSE working solution were equivalent to 116%, 134%, and 147% of those of the control group, respectively, which were consistent with the results of mRNA expression levels (Figure 14).

## 4. Discussion

### 4.1. The Combination of Bioinformatics and Molecular Biology May Be the Key to Opening the “Door” of Understanding the Pathological Process of COPD

Numerous studies have shown that smoking, fuel smoke, air pollution, occupational dusts, infection, chronic bronchitis, and socio-economic status are all inducing factors for COPD. Among them, smoking is the main cause of this disease, and there is a positive correlation between the onset of smoking age, cumulative smoking time, and smoking volume and the incidence of the disease [19,20,21].

It is widely known that COPD is a degenerative disease, which is mainly characterized by airway obstruction and inability of gas to enter and leave the respiratory tract, mainly including chronic bronchitis and emphysema, etc. The pathological process of the disease is closely related to smoking and a variety of environmental factors (such as indoor and outdoor air pollution). Because of this, CSE was used for in vitro experimental validation modeling. In addition, COPD is a disease with obvious genetic characteristics. The genome-wide association studies have screened out several gene loci that are closely related to the occurrence and development of COPD. In view of this, on one hand, it is necessary to intervene in the corresponding environmental risk factors. On the other hand, it is very meaningful to use bioinformatics and molecular biology methods to systematically predict and experimentally verify the factors related to the pathological process of COPD so as to provide scientific reference for the pathogenesis research, clinical diagnosis, and treatment of COPD [22].

### 4.2. Bioinformatics Analysis Showed That Several Molecules Were Closely Related to the Pathological Process of COPD

The reliability of the analysis results is closely related to the preciseness and flexibility of the research strategy. In this study, by referring to the suggested research paradigm available in the literature, the differentially expressed miRNAs were determined according to the intersection of two or more data sets [23]. When the intersection of three data sets was taken at the same time, zero results were screened out. When the intersection of two data sets was taken at the same time, three (hsa-miR-146a, hsa-miR-708, and hsa-miR-150, GSE61741 ∩ GSE136390), one (hsa-miR-454, GSE61741 ∩ GSE38974), and zero (GSE38974 ∩ GSE136390) results were obtained, respectively. The expression levels of hsa-miR-150 and hsa-miR-454 showed the same trends, while hsa-miR-146a and hsa-miR-708 exhibited different trends in the arrays. This result may be related to the temporal and spatial specificities of miRNA expression, which is often encountered in miRNA-related research [24]. Then, the target genes regulated by these four miRNAs were studied.

Sato et al. found that the expression of miR-146a decreased in the lung fibroblasts of COPD patients. Further studies showed that, after the deletion of miR-146a, the expression level of *COX-2*, the target gene regulated by the miRNA, was down-regulated, and then the expression level of prostaglandin E2 (*PGE2*) was up-regulated [25,26,27]. Osei et al. co-cultured human bronchial epithelial cells with primary human lung fibroblasts (from a control population and COPD patients) and then detected the expression of miR-146a-5p. The results showed that the expression level of miR-146a-5p decreased significantly in fibroblasts of COPD patients, and that miR-146a-5p was involved in the functional regulation of lung fibroblasts and negatively regulated epithelia-derived IL-1α induction of IL-8 in fibroblasts. Combined with these results, the authors speculated that the expression level of miR-146a-5p in lung fibroblasts was closely related to the inflammatory phenotype of COPD patients [28]. Keller et al. investigated the whole genome miRNA expression profile of whole blood samples from patients with COPD and carried out survival correlation analysis of the subjects. The results showed that, among a large number of candidate markers, miR-150-5p was differentially expressed between surviving and non-surviving patients. Further analysis showed that miR-150-5p could regulate proliferation-related genes (such as *p53*) [29,30]. The research of Liu et al. showed that the expression level of miR-708-3p was decreased during pulmonary fibrosis and was negatively correlated with idiopathic pulmonary fibrosis (IPF) [31]. Zhu et al. confirmed that miR-454 overexpression was an adverse prognostic factor in patients with non-small cell lung cancer. The down-regulation of miR-454 significantly reduced proliferation, enhanced apoptosis, and inhibited the invasion and migration of non-small cell lung cancer cells [32]. Taken together, the above four miRNAs are closely related to the pathological process of COPD.

In this study, KEGG pathway enrichment analysis showed that the pathways with significant enrichment included TGF-β, PI3K-Akt, ErbB, viral carcinogenesis, non-small cell lung cancer, and the proteoglycan pathway of cancer, etc. Similarly, Liu et al. compared the miRNA expression profiles of COPD patients with healthy controls, and the results showed that miR-23a, miR-25, miR-145, and miR-224 might play important roles in inflammation, oxidative stress, lipid metabolism disorder, cell proliferation, and apoptosis. The enriched pathways included the TGF-β signaling pathway, the p53 signaling pathway, the Wnt signaling pathway, the VEGF signaling pathway, the MAPK signaling pathway, the oxidative stress signaling pathway, and the Notch signaling pathway [33].

It is noteworthy that these signaling pathways are closely related to the pathological process of COPD. For example, studies have shown that the TGF-β signaling pathway plays an important role in the pathogenesis of COPD by regulating cell proliferation, differentiation, extracellular matrix synthesis, and apoptosis. In animal models, an attenuated TGF-β level can cause emphysema. In patients with COPD, TGF-β level is elevated significantly compared with normal controls. TGF-β1, for example, plays an important regulatory role in the pathogenesis of COPD and has a multidirectional effect on adaptive immunity. On one hand, TGF-β1 can regulate T cell response to limit the proliferation of activated T cells and then directly limit the activation of inflammatory Th1 and Th2 by inhibiting T cell proliferation and inducing T cell death. Eventually, it produces anti-inflammatory effects [34,35,36]. On the other hand, TGF-β1 can induce the proliferation of Treg cells, promote the survival of activated T cells, and promote the differentiation of Th17 cells through the Smad and p38 MAPK-dependent intracellular pathways, thus promoting inflammation [37,38]. What is more, the PI3K-Akt signaling pathway plays a key role in regulating cell growth and metabolism. Akt phosphorylated by PI3K can phosphorylate various substrate molecules and regulate a variety of biological effects. Studies have shown that the pulmonary and systemic inflammatory response of COPD can be reduced by regulating the PI3K-Akt signaling pathway. As a key regulator downstream of the PI3K-Akt signaling pathway, FoxO3 plays a key role in the regulation of pneumonia response and antioxidant genes. FoxO3 deficiency can lead to COPD [39,40,41].

### 4.3. The Confirmatory Experimental Results Were Relatively Consistent with the Bioinformatics Analysis Results

The results of the literature search show that there are many methods to simulate the pathogenesis of COPD in vitro. In this experiment, based on the results of relevant studies and our pre-experiments, A549 cells were exposed to 5% CSE working solution to simulate the pathogenesis of COPD. By analyzing the PPI network diagram of COPD-related differentially expressed miRNA target genes, it could be seen that TP53, EP300, and MAPK1 were the key nodes of the protein interaction network. Real-time PCR and ELISA results confirmed that 5% CSE working solution exposure could significantly up-regulate the expression levels of ZEB-1, MAPK1, and EP300, which is relatively consistent with the experimental results in the literature. Sun et al. found that the ZEB-1 level of smokers with COPD was significantly higher than that of smokers and non-smokers without COPD [42]. In vitro studies showed that the level of ZEB-1 in the CSE-exposed group was significantly up-regulated compared with the control group. MAPK can induce a variety of chronic airway inflammatory diseases, including COPD, through oxidative stress and inflammation [43,44]. Rubio et al. showed that the inhibition of EP300 level could improve the fibrosis characteristics in a mouse IPF model (modeled with bleomycin) [45]. Based on this, considering many similarities in the pathogenesis processes between COPD and IPF, it was not difficult to understand that exposure to 5% CSE working solution could significantly increase the expression level of EP300 in A549 cells (as mentioned earlier, this in vitro model was used to simulate the pathological process of COPD). 

Bioinformatics analysis results showed that miR-150 had regulatory relationships with ZEB1 and EP300 and miR-454 had a regulatory relationship with MAPK1. Although miR-146a and miR-708 may not have direct regulatory effects on these three targets, we should still pay attention to the other possible regulatory effects of these two miRNAs on the pathological process of COPD.

In fact, the confirmatory experimental results are not completely consistent with the results of bioinformatics analysis, such as the expression levels of *TP53* and *MYB*, suggesting that the research evidence obtained only by bioinformatics analysis was limited. However, the development of bioinformatics technology is not only a simple analysis of genomic and proteomic data; it can also be the comprehensive analysis of known or new gene products. That being said, bioinformatics as a means of screening for early biomarkers has limitations, in that it only makes predictions based on big data and previous research results, so the accuracy of prediction results cannot be 100%. Because of this, a confirmatory experiment was designed in this study. The combination of bioinformatics analysis and confirmatory experiments may be a better research paradigm for related research.

The incidence of COPD is lower in non-smokers than in smokers. Compared with COPD patients who smoke, non-smoking patients have less impairment in airflow limitation and gas exchange modes and have a lower prevalence of emphysema, chronic cough, etc. It should not be ignored that smoking is only one of the factors leading to COPD. It is necessary to compare the differences in microRNA expression and molecular regulatory networks in COPD caused by different types of exogenous factors.

## 5. Conclusions

**Figure 15:**
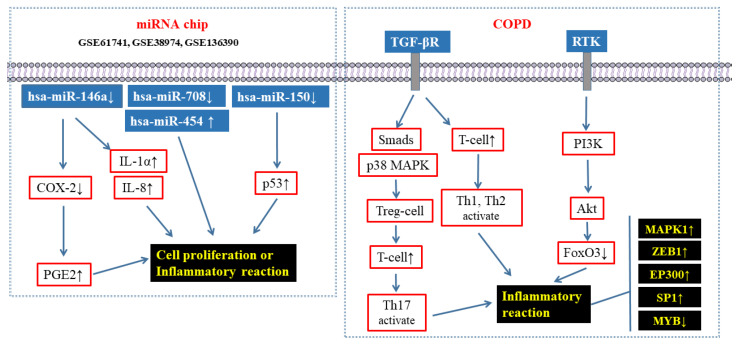
Diagrammatic sketch of the diversified roles of miRNAs and signaling pathways in COPD.

In conclusion, four differentially expressed miRNAs related to COPD (hsa-miR-146a, hsa-miR-708, hsa-miR-150, and hsa-miR-454) were screened out by using the miRNA chip data of COPD in the GEO database. The results of functional enrichment analysis showed that long-term enhancement, the TGF-β signal pathway, and the PI3K-Akt signal pathway were closely related to the pathological process of COPD. PPI analysis showed that ZEB1, EP300, and MAPK1 were the key molecules regulating the pathological process of COPD, which was confirmed by the results of real-time PCR and ELISA. This study provides a theoretical basis for an in-depth understanding of the role of miRNAs and their regulatory targets in the pathological process of COPD and has certain guiding significance for the pathological study, clinical diagnosis, and treatment of the disease. In order to increase readability, a diagrammatic sketch summarizing the various roles of miRNAs and signaling pathways in COPD has been included in Figure 15. 

The main limitation of this study is that only an in vitro cell experiment is used to preliminarily verify the results of bioinformatics analysis. From the perspective of seamless integration between bioinformatics analysis and downstream validation experiments, detecting the expression levels of differentially expressed miRNAs in serum or lung tissue samples of COPD patients (compared to healthy individuals) is more meaningful than conducting in vitro validation experiments. From this perspective, in future research we should consider using the clinical specimens of COPD patients to further verify the research conclusion obtained in this study. Meanwhile, we should also pay attention to the potential regulatory effects of genes that play key roles in the pathological process of COPD on the upstream miRNAs.

## Figures and Tables

**Table 1:** The primer information used in this study.

Symb	Genbank Accession No.	Primer Sequences (5′-3′)
*β-actin*	NM_001101.5	F: GACTACCTCATGAAGATCCTCACC R: TCTCCTTAATGTCACGCACGATT
*EP300*	NM_001362843.2	F: GTTCCTTCCTCAGACTCAGTTC R: CATTATAGGAGAGTTCACCGGG
*MYB*	NM_001130172.2	F: GAAGCAGATTTTTCACCTAGCC R: CTAGGTTCTCCTGCAGGTTTAG
*EGR2*	NM_000399.5	F: CGAATCCACACTGGGCATAAG R: AAACTTTCGGCCACAGTAGTC
*PRKCA*	NM_002737.3	F: GGTGAAGGACCACAAATTCATC R: CACCCGGACAAGAAAAAGTAAC
*TP53*	NM_000546.6	F: TTCCTGAAAACAACGTTCTGTC R: AACCATTGTTCAATATCGTCCG
*MAPK1*	NM_002745.5	F: ATGGTGTGCTCTGCTTATGATA R: TCTTTCATTTGCTCGATGGTTG
*SP1*	NM_001251825.2	F: TCACTCCATGGATGAAATGACA R: CAGAGGAGGAAGAGATGATCTG
*ZEB1*	NM_001128128.3	F: CAGGCAAAGTAAATATCCCTGC R: GGTAAAACTGGGGAGTTAGTCA

## Data Availability

Data are contained within the article.

## Supplementary Materials

The following supporting information can be downloaded at: https://www.mdpi.com/article/10.3390/toxics11120995/s1, Table S1: Differentially expressed miRNAs screened in GSE61741; Table S2: Differentially expressed miRNAs screened in GSE38974; Table S3: Differentially expressed miRNAs screened in GSE136390; Table S4: Quantitative data for PPI analysis.
