# Supplementary material for: Exploration of the Pathogenesis of Chronic Obstructive Pulmonary Disease Caused by Smoking—Based on Bioinformatics Analysis and In Vitro Experimental Evidence
Source: Toxics. 2023 Dec 7;11(12):995. doi: 10.3390/toxics11120995 (PMC10747869; doi:10.3390/toxics11120995)
Supplement: Supplementary file 1 [file toxics-11-00995-s001.zip › toxics-2744097-supplementary.pdf]

Exploration of the pathogenesis of chronic obstructive pulmonary disease caused by smoking - based on bioinformatics analysis and in vitro experimental evidence

Table S1 Differentially expressed miRNAs screened in GSE61741

miRNA ID	*P*. Value	*t*	Fold Change
hsa-miR-186	0.0000254	-7.01396	-5.76439
hsa-miR-512-3p	0.00332997	-3.75357	-4.98003
hsa-miR-323-3p	0.00000241	-9.04048	-4.90256
hsa-miR-1911	0.00085739	-4.5694	-4.20588
hsa-miR-769-5p	0.00849951	-3.21406	-4.19922
hsa-miR-499-5p	0.00040106	-5.0514	-4.10695
hsa-miR-1255b	0.00199905	-4.0546	-4.08811
hsa-miR-208a	0.00128004	-4.32305	-3.95525
hsa-miR-200c	0.04102785	-2.32144	-3.85142
hsa-miR-561	0.00242359	-3.94027	-3.83581
hsa-miR-614	0.03113285	-2.47884	-3.8294
hsa-miR-27a	0.0106894	-3.08386	-3.81711
hsa-miR-1	0.03885861	-2.35252	-3.808
hsa-miR-491-5p	0.00803065	-3.24636	-3.69734
hsa-miR-518b	0.01413329	-2.92576	-3.64701
hsa-miR-657	0.00209782	-4.02588	-3.63514
hsa-miR-431	0.00833233	-3.22536	-3.60055
hsa-miR-300	0.00243549	-3.93737	-3.4712
hsa-miR-1254	0.01131553	-3.0516	-3.46762
hsa-miR-504	0.00251599	-3.91816	-3.46715
hsa-miR-519a	0.00404173	-3.64081	-3.4439
hsa-miR-802	0.00607572	-3.40577	-3.39985
hsa-miR-1252	0.04819781	-2.22896	-3.39717

hsa-miR-526b	0.00252537	-3.91596	-3.39035
hsa-miR-1264	0.0094982	-3.1509	-3.31222
hsa-miR-148b	0.03458335	-2.41903	-3.28052
hsa-miR-337-5p	0.00661079	-3.35743	-3.25833
hsa-miR-122	0.00924597	-3.16619	-3.25268
hsa-miR-549	0.01151488	-3.0417	-3.25185
hsa-miR-369-5p	0.010344	-3.10249	-3.24728
hsa-miR-517b	0.01243398	-2.99822	-3.09424
hsa-miR-577	0.01867111	-2.76838	-3.09041
hsa-miR-518c	0.0121536	-3.01113	-3.08991
hsa-miR-200a	0.02108858	-2.69956	-2.89018
hsa-miR-519d	0.02668692	-2.56629	-2.80153
hsa-miR-548f	0.02735766	-2.55222	-2.79142
hsa-miR-571	0.02546857	-2.59277	-2.77869
hsa-miR-505	0.00639863	-3.3761	-2.77367
hsa-miR-555	0.02747142	-2.54987	-2.64734
hsa-miR-145	0.04620932	-2.25321	-2.5211
hsa-miR-320b	0.0335543	-2.43623	-2.41105
hsa-miR-218-1	0.00141225	-4.26337	-1.96204
hsa-miR-129-3p	0.02425449	-2.62043	-1.84643
hsa-miR-146a	0.00956206	-3.1471	-1.82605
hsa-miR-1273	0.00102541	-4.45877	-1.8007
hsa-miR-181a	0.02178682	-2.68114	-1.59447
hsa-miR-424	0.01503173	-2.89091	-1.59053
hsa-miR-222	0.03413607	-2.42644	-1.57372
hsa-miR-940	0.0150171	-2.89146	-1.57191
hsa-miR-708	0.03752982	-2.3724	-1.55714
hsa-miR-454	0.01083791	-3.07604	-1.49214
hsa-miR-639	0.00378112	-3.67952	-1.48297

hsa-miR-875-3p	0.01363771	-2.94594	-1.42802
hsa-miR-320a	0.01494268	-2.89427	-1.2759
hsa-miR-212	0.03856591	-2.35684	-1.25194
hsa-miR-423-5p	0.02959062	-2.5077	-1.2512
hsa-miR-1539	0.02447112	-2.6154	-1.19216
hsa-miR-938	0.03969399	-2.34036	-1.16935
hsa-miR-487b	0.04022836	-2.33271	-1.16549
hsa-miR-619	0.02304612	-2.64935	-1.09638
hsa-miR-151-5p	0.04396192	-2.28185	-1.03891
hsa-miR-582-3p	0.04294388	-2.29529	-1.03664
hsa-miR-107	0.04413759	-2.27956	-1.03614
hsa-miR-1205	0.03507889	-2.41092	-1.02274
hsa-miR-191	0.04340821	2.28912	1.01564
hsa-miR-20a	0.02908433	2.5175	1.1022
hsa-miR-374b	0.04971338	2.21112	1.15006
hsa-miR-103-as	0.00835606	3.22374	1.19401
hsa-miR-362-3p	0.04946055	2.21406	1.28041
hsa-miR-21	0.01012335	3.11472	1.34113
hsa-miR-1228	0.0447105	2.27216	1.40329
hsa-miR-1282	0.02662069	2.5677	1.42375
hsa-miR-1183	0.00749499	3.28572	1.6494
hsa-miR-196b	0.02218057	2.67101	1.70758
hsa-miR-1268	0.04925273	2.21649	1.80464
hsa-miR-150	0.0417892	2.31091	2.2422
hsa-miR-892b	0.02992748	2.50127	2.68326
hsa-miR-1178	0.04661436	2.24819	3.0375
hsa-miR-1250	0.03411849	2.42674	3.12815
hsa-miR-609	0.03768483	2.37004	3.14176
hsa-miR-649	0.04217521	2.30564	3.65893

hsa-miR-608	0.04565771	2.26011	3.89685
hsa-miR-601	0.01522354	2.88375	3.91936
hsa-miR-125b-1	0.03189772	2.46504	4.00425
hsa-miR-483-3p	0.03029464	2.49435	4.2463
hsa-miR-432	0.01794141	2.79091	4.32925
hsa-miR-924	0.04358246	2.28682	4.3489
hsa-miR-1294	0.03457762	2.41912	4.35228
hsa-miR-509-3p	0.03429698	2.42377	4.53428
hsa-miR-92a-2	0.03719758	2.37747	4.53484
hsa-miR-520d-3p	0.00033675	5.16532	4.85648
hsa-miR-1265	0.00620709	3.3935	4.87651
hsa-miR-23a	0.00145347	4.24596	5.0879
hsa-miR-1182	0.00747057	3.28758	5.18938
hsa-miR-1293	0.00947399	3.15235	5.26636
hsa-miR-30b	0.0075811	3.2792	5.53105
hsa-miR-382	0.01174091	3.03069	5.56834
hsa-miR-187	0.00009454	6.03263	5.75508
hsa-miR-206	0.0003818	5.08335	5.90213

Table S2 Differentially expressed miRNAs screened in GSE38974

**miRNA ID**	***P*.Value**	***t***	**Fold Change**
hsa-miR-223	8.65E-04	-3.718864	-1.5497869
hsa-miR-1274a	1.45E-10	-9.707161	-1.4502034
hsa-miR-105	3.47E-06	-5.732266	-1.2538584
hsa-miR-144	3.42E-03	-3.191856	-1.2501907
hsa-miR-374a	1.96E-04	-4.267951	-1.1717795
hsa-miR-664	7.19E-09	-8.076569	-1.1455395
hsa-miR-148a	1.23E-07	-6.970708	-1.1072064
hsa-miR-454	3.49E-05	-4.894987	-1.0824868
hsa-miR-766	8.44E-06	-5.408915	-1.0433065
hsa-miR-486-5p	3.48E-04	-4.057146	-1.0377131
hsa-miR-10a	8.25E-04	-3.736372	-1.0370846
hsa-miR-25	7.44E-08	7.162854	1.0064691
hsa-miR-513a-5p	3.83E-04	4.021855	1.0413164
hsa-miR-576-3p	9.73E-07	6.198082	1.0458673
hsa-miR-422a	9.82E-07	6.194711	1.0867528
hsa-miR-937	1.75E-06	5.983008	1.2370675
hsa-miR-923	9.71E-06	5.358115	2.3397962

Table S3 Differentially expressed miRNAs screened in GSE136390

miRNA ID	*P.*Value	*t*	Fold Change
hsa-miR-508	0.0002852	-4.7665	-11.0991
hsa-miR-499	0.00013448	-5.1659	-10.8272
hsa-miR-888	0.00003617	-5.892	-10.7229
hsa-miR-220	0.00009246	-5.3692	-10.2322
hsa-miR-525	0.00027523	-4.7852	-10.1867
hsa-miR-522	0.0001323	-5.1747	-8.8391
hsa-miR-302c	0.00054109	-4.4339	-8.7587
hsa-miR-217	0.00052613	-4.4484	-8.6089
hsa-miR-380	0.00406902	-3.4166	-7.9154
hsa-miR-519c	0.00005674	-5.6386	-5.9487
hsa-miR-520d	0.0231197	-2.5439	-5.1456
hsa-miR-346	0.02569676	-2.4896	-5.0467
hsa-miR-302a	0.00423115	-3.3971	-4.6997
hsa-miR-520e	0.00166323	-3.8637	-4.564
hsa-miR-518f	0.00016988	-5.0406	-3.9984
hsa-miR-365	0.00067921	-4.3173	-3.9713
hsa-miR-488	0.04425561	-2.2058	-3.6833
hsa-miR-183	0.02390664	-2.5267	-2.6127
hsa-miR-215	0.02571918	-2.4892	-2.4847
hsa-miR-34c	0.03367085	-2.3496	-2.3463
hsa-miR-143	0.01623968	-2.7238	-1.6454
hsa-miR-582	0.04226817	-2.2302	-1.5657
hsa-miR-192	0.0410484	2.2456	1.0559
hsa-miR-100	0.02744381	2.4557	1.1298
hsa-miR-423	0.03971366	2.2631	1.1817
hsa-miR-130b	0.02449455	2.5142	1.2496
hsa-miR-199a	0.03953347	2.2655	1.3327
hsa-miR-485	0.03423642	2.3409	1.3931

hsa-miR-125b	0.01556013	2.7454	1.4877
hsa-miR-323	0.02303637	2.5457	1.5601
hsa-miR-342	0.01408559	2.7957	1.5711
hsa-miR-95	0.03809234	2.285	1.5719
hsa-miR-146a	0.01366206	2.8111	1.6157
hsa-miR-193b	0.00690796	3.1528	1.6318
hsa-miR-146b	0.01068278	2.9348	1.6434
hsa-miR-141	0.00768493	3.0996	1.6809
hsa-miR-342	0.0156775	2.7416	1.7966
hsa-miR-708	0.0373855	2.2948	1.797
hsa-miR-138	0.0195328	2.63	1.804
hsa-miR-135b	0.02593632	2.4848	1.8633
hsa-miR-29b	0.01207597	2.8733	1.9251
hsa-miR-150	0.00596093	3.2263	1.9328
hsa-miR-154	0.03938708	2.2674	1.9448
hsa-miR-545	0.00953911	2.9916	1.967
hsa-miR-139	0.0020942	3.7482	2.2497
hsa-miR-31	0.00349703	3.4921	2.4512
hsa-miR-337	0.00166186	3.8641	2.7264
hsa-miR-376b	0.03729095	2.2961	2.7373
hsa-miR-493	0.0380032	2.2862	3.357
hsa-miR-184	0.00244203	3.6713	3.7598
hsa-miR-302b	0.00100191	4.1194	4.0133
hsa-miR-642	0.00000464	7.1178	5.4759
hsa-miR-874	0.00000215	7.6127	7.8684

Table S4 Quantitative data for PPI analysis

Node1	Ensembl ID	Node2	Ensembl ID	Interaction score
AIFM2	ENSP00000478931	TP53	ENSP00000269305	0.931
BCAT1	ENSP00000443459	PSAT1	ENSP00000365773	0.541
CBL	ENSP00000264033	CORO1C	ENSP00000394496	0.468
CBL	ENSP00000264033	RAP1B	ENSP00000250559	0.907
CORO1C	ENSP00000394496	CBL	ENSP00000264033	0.468
DDX3X	ENSP00000382840	HNRNPA0	ENSP00000316042	0.455
DDX3X	ENSP00000382840	SP1	ENSP00000329357	0.462
DDX3X	ENSP00000382840	TP53	ENSP00000269305	0.693
EGR2	ENSP00000242480	EP300	ENSP00000263253	0.924
EGR2	ENSP00000242480	HIST2H2BE	ENSP00000358151	0.91
EGR2	ENSP00000242480	Gene homologs	ENSP00000339992	0.418
EGR2	ENSP00000242480	SH3TC2	ENSP00000423660	0.58
EGR2	ENSP00000242480	TP53	ENSP00000269305	0.446
EP300	ENSP00000263253	EGR2	ENSP00000242480	0.924
EP300	ENSP00000263253	HIST2H2BE	ENSP00000358151	0.975
EP300	ENSP00000263253	MAPK1	ENSP00000215832	0.688
EP300	ENSP00000263253	Gene homologs	ENSP00000339992	0.991
EP300	ENSP00000263253	NR2F2	ENSP00000377721	0.735
EP300	ENSP00000263253	PRKCA	ENSP00000408695	0.515
EP300	ENSP00000263253	SP1	ENSP00000329357	0.972
EP300	ENSP00000263253	TP53	ENSP00000269305	0.999
EP300	ENSP00000263253	ZB1	ENSP00000354487	0.644
EPHB2	ENSP00000363763	MAPK1	ENSP00000215832	0.565
EPHB2	ENSP00000363763	TP53	ENSP00000269305	0.654
GOSR1	ENSP00000225724	TP53	ENSP00000269305	0.483
GOSR1	ENSP00000225724	VPS53	ENSP00000401435	0.574
HIST1H2AE	ENSP00000303373	HIST2H2BE	ENSP00000358151	0.789
HIST2H2BE	ENSP00000358151	EGR2	ENSP00000242480	0.91
HIST2H2BE	ENSP00000358151	EP300	ENSP00000263253	0.975
HIST2H2BE	ENSP00000358151	HIST1H2AE	ENSP00000303373	0.789
HIST2H2BE	ENSP00000358151	TP53	ENSP00000269305	0.542
HNRNPA0	ENSP00000316042	DDX3X	ENSP00000382840	0.455
MAPK1	ENSP00000215832	EP300	ENSP00000263253	0.688
MAPK1	ENSP00000215832	EPHB2	ENSP00000363763	0.565
MAPK1	ENSP00000215832	Gene homologs	ENSP00000339992	0.715
MAPK1	ENSP00000215832	PRKCA	ENSP00000408695	0.941
MAPK1	ENSP00000215832	RAP1B	ENSP00000250559	0.937
MAPK1	ENSP00000215832	SP1	ENSP00000329357	0.966
MAPK1	ENSP00000215832	TP53	ENSP00000269305	0.985

MAPK1	ENSP00000215832	ZB1	ENSP00000354487	0.406
MMP14	ENSP00000308208	TP53	ENSP00000269305	0.515
MMP14	ENSP00000308208	ZB1	ENSP00000354487	0.418
Gene homologs	ENSP00000339992	EGR2	ENSP00000242480	0.418
Gene homologs	ENSP00000339992	EP300	ENSP00000263253	0.991
Gene homologs	ENSP00000339992	MAPK1	ENSP00000215832	0.715
Gene homologs	ENSP00000339992	PDCD4	ENSP00000280154	0.552
Gene homologs	ENSP00000339992	TP53	ENSP00000269305	0.955
NR2F2	ENSP00000377721	EP300	ENSP00000263253	0.735
PDCD4	ENSP00000280154	Gene homologs	ENSP00000339992	0.552
PDCD4	ENSP00000280154	TP53	ENSP00000269305	0.714
PDCD4	ENSP00000280154	ZB1	ENSP00000354487	0.477
PRKCA	ENSP00000408695	EP300	ENSP00000263253	0.515
PRKCA	ENSP00000408695	MAPK1	ENSP00000215832	0.941
PRKCA	ENSP00000408695	TP53	ENSP00000269305	0.629
PSAT1	ENSP00000365773	BCAT1	ENSP00000443459	0.541
RAB8A	ENSP00000300935	RAP1B	ENSP00000250559	0.87
RAP1B	ENSP00000250559	CBL	ENSP00000264033	0.907
RAP1B	ENSP00000250559	MAPK1	ENSP00000215832	0.937
RAP1B	ENSP00000250559	RAB8A	ENSP00000300935	0.87
RNF125	ENSP00000217740	TP53	ENSP00000269305	0.406
SH3TC2	ENSP00000423660	EGR2	ENSP00000242480	0.58
SP1	ENSP00000329357	DDX3X	ENSP00000382840	0.462
SP1	ENSP00000329357	EP300	ENSP00000263253	0.972
SP1	ENSP00000329357	MAPK1	ENSP00000215832	0.966
SP1	ENSP00000329357	TP53	ENSP00000269305	0.987
SP1	ENSP00000329357	ZBTB7A	ENSP00000323670	0.508
TP53	ENSP00000269305	AIFM2	ENSP00000478931	0.931
TP53	ENSP00000269305	DDX3X	ENSP00000382840	0.693
TP53	ENSP00000269305	EGR2	ENSP00000242480	0.446
TP53	ENSP00000269305	EP300	ENSP00000263253	0.999
TP53	ENSP00000269305	EPHB2	ENSP00000363763	0.654
TP53	ENSP00000269305	GOSR1	ENSP00000225724	0.483
TP53	ENSP00000269305	HIST2H2BE	ENSP00000358151	0.542
TP53	ENSP00000269305	MAPK1	ENSP00000215832	0.985
TP53	ENSP00000269305	MMP14	ENSP00000308208	0.515
TP53	ENSP00000269305	Gene homologs	ENSP00000339992	0.955

TP53	ENSP00000269305	PDCD4	ENSP00000280154	0.714
TP53	ENSP00000269305	PRKCA	ENSP00000408695	0.629
TP53	ENSP00000269305	RNF125	ENSP00000217740	0.406
TP53	ENSP00000269305	SP1	ENSP00000329357	0.987
TP53	ENSP00000269305	ZBTB7A	ENSP00000323670	0.769
TP53	ENSP00000269305	ZB1	ENSP00000354487	0.665
VPS53	ENSP00000401435	GOSR1	ENSP00000225724	0.574
ZBTB7A	ENSP00000323670	SP1	ENSP00000329357	0.508
ZBTB7A	ENSP00000323670	TP53	ENSP00000269305	0.769
ZB1	ENSP00000354487	EP300	ENSP00000263253	0.644
ZB1	ENSP00000354487	MAPK1	ENSP00000215832	0.406
ZB1	ENSP00000354487	MMP14	ENSP00000308208	0.418
ZB1	ENSP00000354487	PDCD4	ENSP00000280154	0.477
ZB1	ENSP00000354487	TP53	ENSP00000269305	0.665
